# Entropy-stabilized oxides

**DOI:** 10.1038/ncomms9485

**Published:** 2015-09-29

**Authors:** Christina M. Rost, Edward Sachet, Trent Borman, Ali Moballegh, Elizabeth C. Dickey, Dong Hou, Jacob L. Jones, Stefano Curtarolo, Jon-Paul Maria

**Affiliations:** 1 Department of Materials Science and Engineering, North Carolina State University, Raleigh, North Carolina 27695, USA; 2 Department of Mechanical Engineering and Materials Science, Center for Materials Genomics, Duke University, Durham, North Carolina 27708, USA

## Abstract

Configurational disorder can be compositionally engineered into mixed oxide by populating a single sublattice with many distinct cations. The formulations promote novel and entropy-stabilized forms of crystalline matter where metal cations are incorporated in new ways. Here, through rigorous experiments, a simple thermodynamic model, and a five-component oxide formulation, we demonstrate beyond reasonable doubt that entropy predominates the thermodynamic landscape, and drives a reversible solid-state transformation between a multiphase and single-phase state. In the latter, cation distributions are proven to be random and homogeneous. The findings validate the hypothesis that deliberate configurational disorder provides an orthogonal strategy to imagine and discover new phases of crystalline matter and untapped opportunities for property engineering.

## Abstract (web summary)

The composition of oxide compounds controls many of their properties and electronic phases. Here, the authors show that entropy and configurational disorder can stabilize new phases of oxides, potentially enabling a better engineering of their properties.

## Graphical abstract

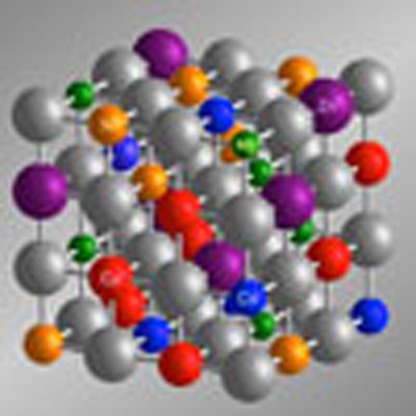

A grand challenge facing materials science is the continuous hunt for advanced materials with properties that satisfy the demands of rapidly evolving technology needs. The materials research community has been addressing this problem since the early 1900s when Goldschmidt reported the ‘the method of chemical substitution'^1^ that combined a tabulation of cationic and anionic radii with geometric principles of ion packing and ion radius ratios. Despite its simplicity, this model enabled a surprising capability to predict stable phases and structures. As early as 1926 many of the technologically important materials that remain subjects of contemporary research were identified (though their properties were not known); BaTiO_3_, AlN, GaP, ZnO and GaAs are among that list.

These methods are based on overarching natural tendencies for binary, ternary and quaternary structures to minimize polyhedral distortions, maximize space filling and adopt polyhedral linkages that preserve electroneutrality^1^,^2^,^3^. The structure-field maps compiled by Muller and Roy catalogue the crystallographic diversity in the context of these largely geometry-based predictions^4^. There are, however, limitations to the predictive power, particularly when factors like partial covalency and heterodesmic bonding are considered.

To further expand the library of advanced materials and property opportunities, our community explores possibilities based on mechanical strain^5^, artificial layering^6^, external fields^7^, combinatorial screening^8^, interface engineering^9^,^10^ and structuring at the nanoscale^6^,^11^. In many of these efforts, computation and experiment are important companions.

Most recently, high-throughput methods emerged as a powerful engine to assess huge sections of composition space^12^,^13^,^14^,^15^,^16^,^17^ and identified rapidly new Heusler alloys, extensive ion substitution schemes^18^,^19^, new 18-electron ABX compounds^20^ and new ferroic semiconductors^21^.

While these methods offer tremendous predictive power and an assessment of composition space intractable to experiment, they often utilize density functional theory calculations conducted at 0 K. Consequently, the predicted stabilities are based on enthalpies of formation. As such, there remains a potential section of discovery space at elevated temperatures where entropy predominates the free-energy landscape.

This landscape was explored recently by incorporating deliberately five or more elemental species into a single lattice with random occupancy. In such crystals, entropic contributions to the free energy, rather than the cohesive energy, promote thermodynamic stability at finite temperatures. The approach is being explored within the high-entropy-alloy family of materials (HEAs)^22^, in which extremely attractive properties continue to be found^23^,^24^. In HEAs, however, discussion remains regarding the true role of configurational entropy^25^,^26^,^27^,^28^, as samples often contain second phases, and there are uncertainties regarding short-range order. In response to these open discussions, HEAs have been referred to recently as multiple-principle-element alloys^29^.

It is compelling to consider similar phenomena in non-metallic systems, particularly considering existing information from entropy studies in mixed oxides. In 1967 Navrotsky and Kleppa showed how configurational entropy regulates the normal-to-inverse transformation in spinels, where cations transition between ordered and disordered site occupancy among the available sublattices^30^,^31^. These fundamental thermodynamic studies lead one to hypothesize that in principle, sufficient temperature would promote an additional transition to a structure containing only one sublattice with random cation occupancy. From experiment we know that before such transitions, normal materials melt, however, it is conceivable that synthetic formulations exist, which exhibit them.

Inspired by research activities in the metal alloy communities and fundamental principles of thermodynamics we extend the entropy concept to five-component oxides. With unambiguous experiments we demonstrate the existence of a new class of mixed oxides that not only contains high configurational entropy but also is indeed truly entropy stabilized. In addition, we present a hypothesis suggesting that entropy stabilization is particularly effective in a compound with ionic character.

## Results

### Choosing an appropriate experimental candidate

The candidate system is an equimolar mixture of MgO, CoO, NiO, CuO and ZnO, (which we label as ‘E1') so chosen to provide the appropriate diversity in structures, coordination and cationic radii to test directly the entropic *ansatz*. The rationale for selection is as follows: the ensemble of binary oxides should not exhibit uniform crystal structure, electronegativity or cation coordination, and there should exist pairs, for example, MgO–ZnO and CuO–NiO, that do not exhibit extensive solubility. Furthermore, the entire collection should be isovalent such that relative cation ratios can be varied continuously with electroneutrality preserved at the net cation to anion ration of unity. Tabulated reference data for each component, including structure and ionic radius, can be found in Supplementary Table 1.

### Testing reversibility

**Figure 1:**
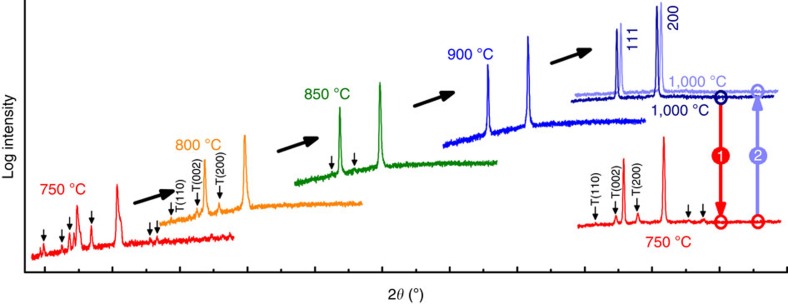
X-ray diffraction patterns for entropy-stabilized oxide formulation E1. E1 consists of an equimolar mixture of MgO, NiO, ZnO, CuO and CoO. The patterns were collected from a single pellet. The pellet was equilibrated for 2 h at each temperature in air, then air quenched to room temperature by direct extraction from the furnace. X-ray intensity is plotted on a logarthimic scale and arrows indicate peaks associated with non-rocksalt phases, peaks indexed with (T) and with (RS) correspond to tenorite and rocksalt phases, respectively. The two X-ray patterns for 1,000 °C annealed samples are offset in 2*θ* for clarity.

In the first experiment, ceramic pellets of E1 are equilibrated in an air furnace and quenched to room temperature. The temperature spanned a range from 700 to 1,100 °C, in 50-°C increments. X-ray diffraction patterns showing the phase evolution are depicted in Fig. 1. After 700 °C, two prominent phases are observed, rocksalt and tenorite. The tenorite phase fraction reduces with increasing equilibration temperature. Full conversion to single-phase rocksalt occurs between 850 and 900 °C, after which there are no additional peaks, the background is low and flat, and peak widths are narrow in two-theta (2*θ*) space.

Reversibility is a requirement of entropy-driven transitions. Consequently, low-temperature equilibration should transform homogeneous 1,000 °C-equilibrated E1 back to its multiphase state (and vice versa on heating). Figure 1 also shows a sequence of X-ray diffraction patterns for such a thermal excursion; initial equilibration at 1,000 °C, a second anneal at 750 °C, and finally a return to 1,000 °C. The transformation from single phase, to multiphase, to single phase is evident by the X-ray patterns and demonstrates an enantiotropic (that is, reversible with temperature^32^) phase transition.

### Testing entropy though composition variation

A composition experiment is conducted to further characterize this phase transition to the random solid solution state. If the driving force is entropy, altering the relative cation ratios will influence the transition temperature. Any deviation from equimolarity will reduce the number of possible configurations Ω (*S*_c_=*k*_B_log(Ω)), thus increasing the transition temperature. Because *S*_c_(*x*_*i*_) is logarithmically linked to mole fraction via ∼*x*_*i*_log(*x*_*i*_), the compositional dependence is substantial.

This dependency underpins our *gedankenexperiment* where the role of entropy can be tested by measuring the dependency of transition temperature as a function of the total number of components present, and of the composition of a single component about the equimolar formulation.

**Figure 2:**
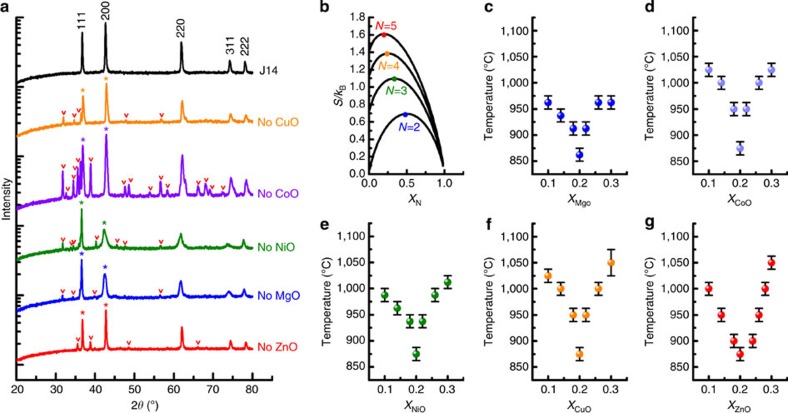
Compositional analysis. (**a**) X-ray diffraction analysis for a composition series where individual components are removed from the parent composition E1 and heat treated to the conditions that would otherwise produce full solid solution. Asterisks identify peaks from rocksalt while carrots identify peaks from other crystal structures. (**b**) Calculated configurational entropy in an *N*-component solid solutions as a function of mol% of the *N*^th^ component, and (**c**–**g**) partial phase diagrams showing the transition temperature to single phase as a function of composition (*solvus*) in the vicinity of the equimolar composition where maximum configurational entropy is expected. Error bars account for uncertainty between temperature intervals. Each phase diagram varies systematically the concentration of one element.

The calculated entropy trends for an ideal mixture are illustrated in Fig. 2b, which plots configurational entropy for a set of mixtures having *N* species where the composition of an individual species is changed and the others (*N*−1) are kept equimolar. Two dependencies become apparent: the entropy increases as new species are added and the maximum entropy is achieved when all the species have the same fraction. Both dependencies assume ideal random mixing. Two series of composition-varying experiments investigate the existence of these trends in formulation E1.

The first experiment monitors phase evolution in five compounds, each related to the parent E1 by the extraction of a single component. The sets are equilibrated at 875 °C (the threshold temperature for complete solubility) for 12 h. The diffraction patterns in Fig. 2a show that removing any component oxide results in material with multiple phases. A four-species set equilibrated under these conditions never yields a single-phase material.

The second experiment uses five individual phase diagrams to explore the configurational entropy versus composition trend. In each, the composition of a single component is varied by ±2, ±6 and ±10% increments about the equimolar composition while the others are kept even. Since any departure from equimolarity reduces the configurational entropy, it should increase transition temperatures to single phase, if that transition is in fact entropy driven. The specific formulations used are given in Supplementary Table 2.

Figure 2c–g are phase diagrams of composition versus transformation temperature for the five sample sets that varied mole fraction of a single component. The diagrams were produced by equilibrating and quenching individual samples in 25 °C intervals between 825 and 1,125 °C to obtain the *T*_trans_-composition *solvus*. In all cases equimolarity always leads to the lowest transformation temperatures. This is in agreement with entropic promotion, and consistent with the ideal model shown in Fig. 2b. One set of raw X-ray patterns used to identify *T*_trans_ for 10% MgO is given as an example in Supplementary Fig. 1.

### Testing endothermicity

**Figure 3:**
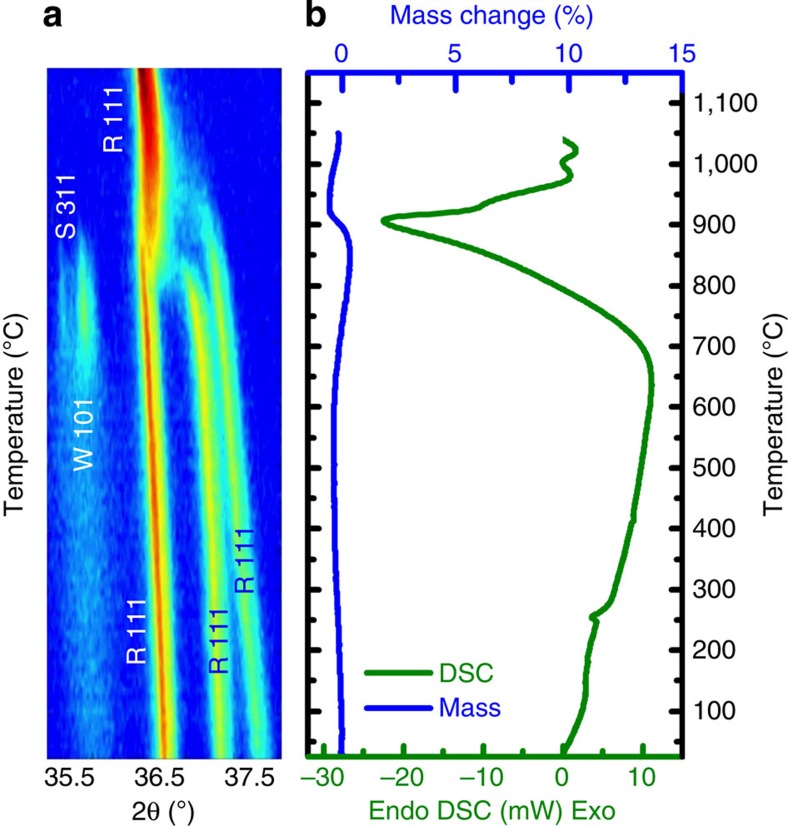
Demonstrating endothermicity. (**a**) *In situ* X-ray diffraction intensity map as a function of 2*θ* and temperature; and (**b**) differential scanning calorimetry trace for formulation ‘E1'. Note that the conversion to single phase is accompanied by an endotherm. Both experiments were conducted at a heating rate of 5 °C min^−1^.

Reversibility and compositionally dependent *solvus* lines indicate an entropy-driven process. As such, the excursion from polyphase to single phase should be endothermic. An entropy-driven solid–solid transformation is similar to melting, thus requires heat from an external source^33^. To test this possibility, the phase transformation in formulation E1 can be co-analysed with differential scanning calorimetry and *in situ* temperature-dependent X-ray diffraction using identical heating rates. The data for both measurements are shown in Fig. 3. Figure 3a is a map of diffracted intensity versus diffraction angle (abscissa) as a function of temperature. It covers ∼4° of 2*θ* space centred about the 111 reflection for E1. At a temperature interval between 825 and 875 °C, there is a distinct transition to single-phase rocksalt structure—all diffraction events in that range collapse into an intense <111> rocksalt peak.

Figure 3b contains the companion calorimetric result where one finds a pronounced endotherm in the identical temperature window. The endothermic response only occurs when the system adds heat to the sample, uniquely consistent with an entropy-driven transformation^33^. We note the small mass loss (∼1.5%) at the endothermic transition. This mass loss results from the conversion of some spinel (an intermediate phase seen by X-ray diffraction) to rocksalt, which requires reduction of 3^+^ to 2^+^ cations and release of oxygen to maintain stoichiometry. To address concerns regarding CuO reduction, Supplementary Fig. 2 shows a differential scanning calorimetry and thermal gravimetric analysis curve for pure CuO collected under the same conditions. There is no oxygen loss in the vicinity of 875 °C.

### Testing homogeneity

All experimental results shown so far support the entropic stabilization hypothesis. However, all assume that homogeneous cation mixing occurs above the transition temperature. It is conceivable that local composition fluctuations produce coherent clustering or phase separation events that are difficult to discern by diffraction using a laboratory sealed tube diffractometer. The *solvus* lines of Fig. 2c–g support random mixing, as the most stable composition is equimolar (a condition only expected for ideal/regular solutions), but it is appropriate to ensure self-consistency with direct measurements. To characterize the cation distributions, extended X-ray absorption fine structure (EXAFS) and scanning transmission electron microscopy with energy dispersive X-ray spectroscopy (STEM EDS) is used to analyse structure and chemistry on the local scale.

**Figure 4:**
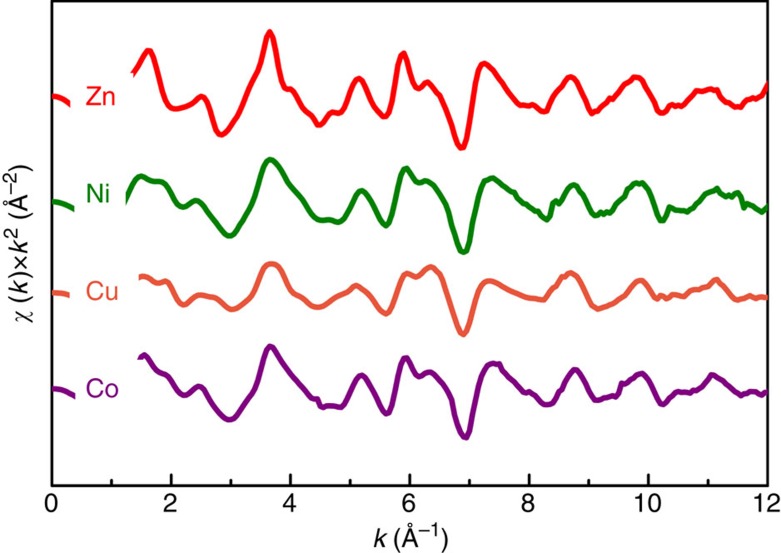
Extended X-ray absorption fine structure. EXAFS measured at Advanced Photon Source beamlime 12-BM after energy normalization and fitting. Note that the oscillations for each element occur with similar relative intensity and at similar reciprocal spacing. This suggests a similar local structural and chemical environment for each.

EXAFS data were collected for Zn, Ni, Cu and Co at the Advanced Photon Source 12-BM-B^34^,^35^. The fitted data are shown in Fig. 4, the raw data are given in Supplementary Fig. 3. The fitted data for each element provide two conclusions: the cation-to-anion first-near-neighbour distances are identical (within experimental error of ±0.01 Å) and the local structures for each element to approximately seven near-neighbour distances are similar. Both observations are only consistent with a random cation distribution.

**Figure 5:**
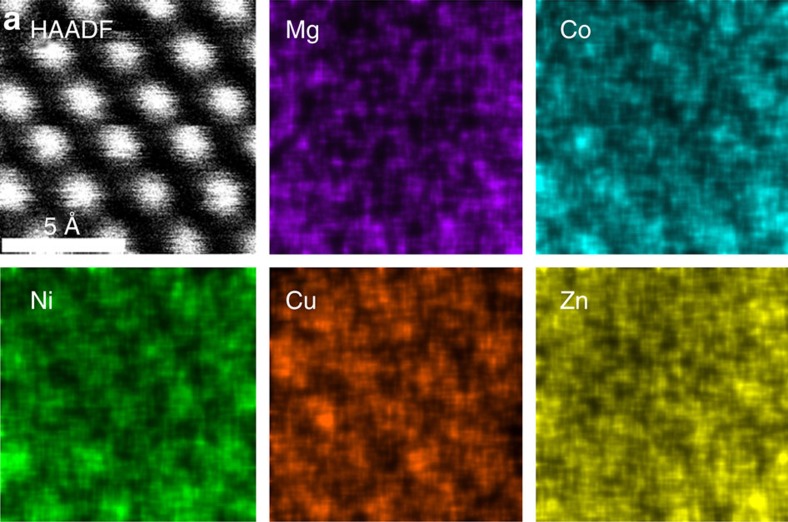
STEM–EDS analysis of E1. (**a**) HAADF image. Panels labelled as Zn, Ni, Cu, Mg and Co are intensity maps for the respective characteristic X-rays. The individual EDS maps show uniform spatial distributions for each element and are atomically resolved.

As a corroborating measure of local homogeneity, chemical analysis was conducted using a probe-corrected FEI Titan STEM with EDS detection. Thin film samples of E1, prepared by pulsed laser deposition, are the most suitable samples to make the assessment. Details of preparation are given in the methods, and X-ray and electron diffraction analysis for the film are provided in Supplementary Figs 4 and 5. The sample was thinned by mechanical polishing and ion milling. Figure 5 shows a collection of images including Fig. 5a, the high-angle annular dark-field signal (HAADF).

In Fig. 5b–f, the EDS signals for the Kα emission energies of Mg, Co, Ni, Cu and Zn are shown (additional lower magnification images are included in Supplementary Fig. 6). All magnifications reveal chemically and structurally homogeneous material.

X-ray diffraction, EXAFS and STEM–EDS probes are sensitive to 10 s of nm, 10 s of Å and 1 Å length scales, respectively. While any single technique could be misinterpreted to conclude homogenous mixing, the combination of X-ray diffraction, EXAFS and STEM–EDS provide very strong evidence. We note, in particular, the similarity in EXAFS oscillations (both in amplitude and position) out to 12 inverse angstroms. This similarly would be lost if local ordering or clustering were present. Consequently, we conclude with certainty that the cations are uniformly dispersed.

## Discussion

The set of experimental outcomes show that the transition from multiple-phase to single phase in E1 is driven by configurational entropy. To complete our thermodynamic understanding of this system, it is important to understand and appreciate the enthalpic penalties that establish the transition temperature. In so doing, the data set can be tested for self-consistency, and the present data are brought into the context of prior research on oxide solubility.

First, we consider an equation relating the initial and final states of the proposed phase transition:


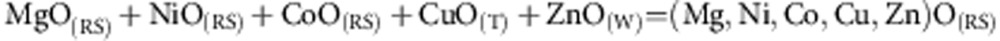


For MgO, NiO and CoO, the crystal structures of the initial and final states are identical. If we assume that solution of each into the E1 rocksalt phase is ideal, the enthalpy for mixing is zero. For CuO and ZnO, there must be a structural transition to rocksalt on dissolution from tenorite and wurtzite, respectively. If we again assume (for simplicity) that the solution is ideal, the mixing energy is zero, but there is an enthalpic penalty associated with the structure transition. From Davies *et al*. and Bularzik *et al*., we know the reference chemical potential changes for the wurtzite-to-rocksalt and the tenorite-to-rocksalt transitions of ZnO and CuO; they are 25 and 22 kJ mol^−1^, respectively^36^,^37^. If we make the assumption that the transition enthalpies of ZnO(wurtzite) to ZnO(rocksalt E1) and CuO(tenorite) to CuO(rocksalt E1) are comparable, then the enthalpic penalty for solution into E1 can be estimated. For ZnO and CuO, the transition to solid solution in a rocksalt structure involves an enthalpy change of (0.2)·(25 kJ mol^−1^)+(0.2)·(22 kJ mol^−1^), a total of +10 kJ mol^−1^. This calculation is based on the product of the mol fraction of each multiplied by the reference transition enthalpy.

This assumption is consistent with the report of Davies *et al*. who showed that the chemical potential of a particular cation in a particular structure is associated with the molar volume of that structure^36^. Since the rocksalt phases of ZnO and CuO have molar volumes comparable to E1, their reference transition enthalpy values are considered suitable proxies.

In comparison, the maximum theoretically expected configurational entropy difference at 875 °C (the temperature were we observe the transition experimentally) between the single species and the random five-species solid solution is ∼15 kJ mol^−1^, 5 kJ mol^−1^ larger than the calculated enthalpy of transition. It is possible that the origins of this difference are related to mixing energy as the reference energy values for structural transitions to rocksalt do not capture that aspect.

While the present phase diagrams that monitor *T*_trans_ as a function of composition demonstrate rather symmetric behaviour about the temperature minima, it is unlikely that mixing enthalpies are zero for all constituents. Indeed, literature reports show that enthalpies of mixing between the constituent oxides in E1 are finite and of mixed sign, and their magnitudes are on the same order as the 5 kJ mol^−1^ difference between our calculated predictions^36^. This energy difference may be accounted for by finite and positive mixing enthalpies.

Following this argument, we can achieve a self-consistent appreciation for the entropic driving force and the enthalpic penalties for solution formation in E1 by considering enthalpies of the associated structural transitions and expected entropy values for ideal cation mixing.

As a final test, these predictions can be compared with experiment, specifically by calculating the magnitude of the endotherm observed by DSC at the transition from multiple-phase to single-phase states. Doing so we find a value ∼12 kJ mol^−1^ (with an uncertainty of ±2 kJ mol^−1^). While we acknowledge the challenge of quantitative calorimetry, we note that this experimental result is intermediate to and in close agreement with the predicted values.

Compared with metallic alloys, the pronounced impact of entropy in oxides may be surprising given that on a per-atom basis the total disorder per volume of an oxide seems be lower than in a high-entropy alloy, as the anion sublattice is ordered (apart from point defects). The chemically uniform sublattice is perhaps the key factor that retains cation configurational entropy. As an illustration, consider a comparison between random metal alloys and random metal oxide alloys.

**Figure 6:**
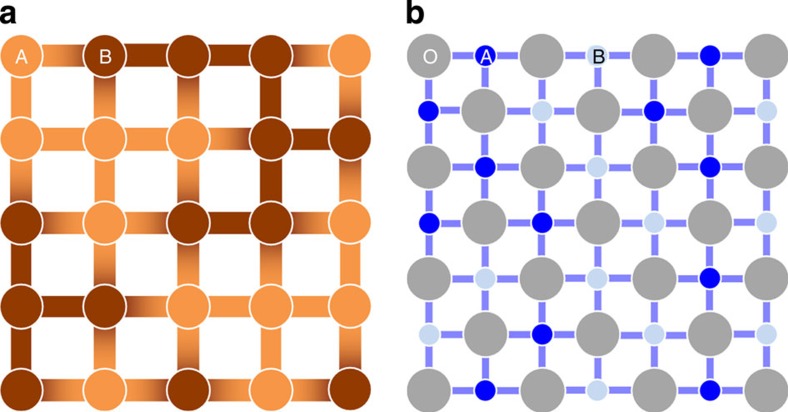
Binary metallic compared with a ternary oxide. A schematic representation of two lattices illustrating how the first-near-neighbour environments between species having different electronegativity (the darker the more negative charge localized) for (**a**) a random binary metal alloy and (**b**) a random pseudo-binary mixed oxide. In the latter, near-neighbour cations are interrupted by intermediate common anions.

Begin by reviewing the case of a two-component metallic mixture A–B. If the mixture is ideal, the energy of interaction *E*_A–B_=(*E*_A–A_+*E*_B–B_)/2, there is no enthalpic preference for bonding, and entropy regulates solution formation. In this scenario, all lattice sites are equivalent and configurational entropy is maximized. This situation, however, never occurs as no two elements have identical electronegativity and radii values. Figure 6a illustrates a two-component alloy scenario A–B where species B is more electronegative than A. Consequently, the interaction energies *E*_A–A_, *E*_B–B_ and *E*_A–B_ will be different. A random mixture of A–B will produce lattice sites with a distribution of first near neighbours, that is, species A coordinated to 4-B atoms, 2-A and 2-B atoms, etc… Different coordinations will have different energy values and the sites are no longer indistinguishable. Reducing the number of equivalent sites reduces the number of possible configurations and *S*.

Now consider the same two metallic ions co-populating a cation sublattice, as in Fig. 6b. In this case, there is always an intermediate anion separating neighbouring cation lattice sites. Again, in the limiting case where only first near neighbours are considered, every cation lattice site is ‘identical' because each has the same immediate surroundings: the interior of an oxygen octahedron. Differentiation between sites is only apparent when the second near neighbours are considered. From the configurational disorder perspective, if each cation lattice site is identical, and thus energetically similar to all others, the number of microstates possible within the macrostate will approach the maximum value.

This crystallographic argument is based on the limiting case where first-near-neighbour interactions predominate the energy landscape, which is an imperfect approximation. Second and third near neighbours will influence the distribution of lattice site energies and the number of equivalent microstates—but the impact will be the same in both scenarios. A larger number of equivalent sites in a crystal with an intermediate sublattice will increase *S* and expand the elemental diversity containable in a single solid solution and to lower the temperature at which the transition to entropic stabilization occurs. We acknowledge the hypothesis nature of this model at this time, and the need for a rigorous theoretical exploration. It is presented currently as a possibility and suggestion for future consideration and testing.

We demonstrate that configurational disorder can promote reversible transformations between a poly-phase mixture and a homogeneous solid solution of five binary oxides, which do not form solid solutions when any of the constituents are removed provided the same thermal budget. The outcome is representative of a new class of materials called ‘entropy-stabilized oxides'. While entropic effects are known for oxide systems, for example, random cation occupancy in spinels^30^, order–disorder transformations in feldspar^38^, and oxygen nonstoichiometry in layered perovskites^39^, the capacity to actively engineer configurational entropy by composition, to stabilize a quinternary oxide with a single cation sublattice, and to stabilize unusual cation coordination values is new. Furthermore, these systems provide a unique opportunity to explore the thermodynamics and structure–property relationships in systems with extreme configurational disorder.

Experimental efforts exploring this composition space are important considering that such compounds will be challenging to characterize with computational approaches minimizing formation energy (for example, genetic algorithms) or with *adhoc* thermodynamic models (for example, CALPHAD, cluster expansion)^6^.

We expect entropic stabilization in systems where near-neighbour cations are interrupted by a common intermediate anion (or vice versa), which includes broad classes of chalcogenides, nitrides and halides; particularly when covalent character is modest. The entropic driving force—engineered by cation composition—provides a departure from traditional crystal-chemical principles that elegantly predict structural trends in the major ternary and quaternary systems. A companion set of structure–property relationships that predict new entropy-stabilized structures with novel cation incorporation await discovery and exploitation.

## Methods

### Solid-state synthesis of bulk materials

MgO (Alfa Aesar, 99.99%), NiO (Sigma Aldrich, 99%), CuO (Alfa Aesar, 99.9%), CoO (Alfa Aesar, 99%) and ZnO (Alfa Aesar 99.9%) are massed and combined using a shaker mill and 3-mm diameter yttrium-stabilized zirconia milling media. To ensure adequate mixing, all batches are milled for at least 2 h. Mixed powders are then separated into 0.500-g samples and pressed into 1.27-cm diameter pellets using a uniaxial hydraulic press at 31,000 N. The pellets are fired in air using a Protherm PC442 tube furnace.

### Temperature evolution of phases

Ceramic pellets of E1 are equilibrated in an air furnace and quenched to room temperature by direct extraction from the hot zone. Phase analysis is monitored by X-ray diffraction using a PANalytical Empyrean X-ray diffractometer with Bragg-Brentano optics including programmable divergence and receiving slits to ensure constant illumination area, a Ni filter, and a 1-D 128 element strip detector. The equivalent counting time for a conventional point detector would be 30 s per point at 0.01° 2*θ* increments. Note that all X-ray are collected using substantial counting times and are plotted on a logarithmic scale. To the extent knowable using a laboratory diffractometer, the high-temperature samples are homogeneous and single phase: there are no additional minor peaks, the background is low and flat, and peak widths are sharp in two-theta (2*θ*) space.

Temperature-dependent diffraction data are collected with PANalytical Empyrean X-ray diffractometer with Bragg-Brentano optics including programmable divergence and receiving slits to ensure constant illumination area, a Ni filter, and a 1-D 256 element strip detector. The samples are placed in a resistively heated HTK-1200N hot stage in air. The samples are ramped at a constant rate of 5 °C min^−1^ with a theta–two theta pattern captured every 1.5 min. Calorimetry data are collected using a Netzsch STA 449 F1 Jupiter system in a Pt crucible at 5 °C min^−1^ in flowing air.

### Determining *solvus* lines

Five series of powders are mixed where the amount of one constituent oxide is varied from the parent mixture E1. Supplementary Table 2 lists the full set of samples synthesized for this experiment. Each individual sample is cycled through a heat-soak-quench sequence at 25 °C increments from 850 °C up to 1,150 °C. The soak time for each cycle is 2 h, and samples are then quenched to room temperature in <1 min.

After the quenching step for each cycle, samples are immediately analysed for phase identification using a PANalytical Empyrean X-ray diffractometer using the conditions identified above. If more than one phase is present, the sample would be put through the next temperature cycle. The temperature at which the structure is determined to be pure rocksalt, with no discernable evidence of peak splitting or secondary phases, is deemed the transition temperature as a function of composition. Supplementary Fig. 1 shows an example of the collected X-ray patterns after each cycle using the E1L series with +10% MgO. Once single phase is achieved, the sample is removed from the sequence.

Note that this entire experiment is conducted two times. Initially in 50 °C increments and longer anneals, and to ensure accuracy of temperature values and reproducibility, a second time using shorter increments and 25 °C anneals. Findings in both sets are identical to within experimental error bar values. In the latter case, error bars correspond to the annealing interval value of 25 °C.

In the main text relating to Fig. 2a we note that in addition to small peaks from second phases, X-ray spectra for *N*=4 samples with either NiO or MgO removed show anisotropic peak broadening in 2*θ* and skewed relative intensities where *I*_(200)_/*I*_(111)_ is less than unity. This ratio is not possible for the rocksalt structure. Supplementary Table 3 shows the result of calculations of structure factors for a random equimolar rocksalt oxide with composition E1. Calculations show that the 200 reflection is the strongest, and that the experimentally measured relative intensities of 111/200 are consistent with calculations. We use this information as a means too best assess when the transition to single phase occurs since the most likely reason for the skewed relative intensity is an incomplete conversion to the single-phase state. This dependency is highlighted in Supplementary Fig. 1.

### X-ray absorption fine structure

X-ray absorption fine structure (XAFS) is made possible through the general user programme at the Advanced Photon Source in Lemont, IL (GUP-38672). This technique provides a unique way to probe the local environment of a specific element based on the interference between an emitted core electron and the backscattering from surrounding species. XAFS makes no assumption of structure symmetry or elemental periodicity, making it an ideal means to study disordered materials. During the absorption process, core electrons will absorb incident X-ray energies equal to or greater than their respective binding energies. The emitted photoelectron wave interacts with neighbouring species, and the resulting absorption spectrum, displayed as absorption intensity versus incident energy, shows characteristic modulations unique to the target atom and its environment.

Equimolar amounts of the constituent oxides (MgO, NiO, CuO, CoO and ZnO) are mixed and pre-reacted at 1,000 °C in air for 12 h with intermittent stirring during calcination. The product is mixed into an isopropyl alcohol slurry and ball milled using yttrium-stabilized media for 24 h. The powder is then dried in a fume hood at room temperature then re-fired at 1,000 °C for 12 h, then checked via X-ray diffraction to ensure phase purity and that peaks remain narrow and intense. Milled grain size is measured using scanning electron microscopy and determined to average ∼10 μm.

A 2:1 powder to 10% PVA/H_2_O suspension is mixed continuously to disperse particles within the solution as well as aid in breaking up any agglomerates. Using a Cookson Electronics P-6000 spin coater, thin layers are spun onto 2 × 2 cm square pieces of 25-μm thick Kapton. By trial and observation, it is determined that spinning at 2,000 r.p.m. for 1 min makes a homogenous thin film with the appropriate quantity of particles for XAFS analysis.

Advanced Photon Source beamline 12-BM is utilized for its energy range of 4.5–23 keV, which can probe all cation species except magnesium. Absorption spectra are recorded as a function of energy using a fluorescence set-up^40^ with a Canberra 13-element Ge detector. The energies per measurement range from 150 eV before the known K absorption edge of the target element to ∼1,000 eV past the edge onset. Supplementary Table 3 lists the cation species of interest and their respective K edges. Simultaneously to the sample fluorescence, reference foils are measured in transmission mode. This enables the energy calibration of the data relative to the theoretical edge of the metal, since compounds tend to have a slight variation in their absorption-edge energies. Each measurement is repeated three times to check for systematic error and to improve signal to noise ratio.

The raw data shown in Supplementary Fig. 2a plots the absorption edge and modulations on the post edge background. In order to isolate the EXAFS from these spectra, a background function is fit and subtracted. Energy space is transformed into *k*-space via the equation^25^:


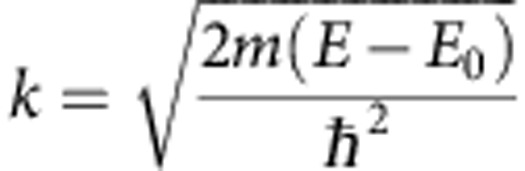


where *m* is electron mass, *ħ* is the reduced Planck's constant, and *E*_0_ is the absorption-edge energy. Supplementary Fig. 2b shows the isolated EXAFS from the measurement. With this data, qualitative conclusions can be made pertaining to the degree of randomness of the cation species. If there were ordering within the system, these spectra would not demonstrate such consistent oscillatory structure and the scattering pathways for individual species would be unique. We limit our current conclusions at this somewhat conservative level as it provides the evidence needed to support a random solid solution.

### Scanning transmission electron microscopy

To best facilitate sample preparation and atomic-resolution analysis in STEM, a single crystal E1 thin film is grown on a {100} MgO substrate using pulsed laser deposition and thinned to electron transparency. The deposition process used a KrF 248 nm excimer laser; with an energy density of 3 J cm^−2^, substrate temperature of 600 °C, an oxygen pressure of 50 mtorr and target to substrate distance of 4 cm. A deposition rate of 6 Hz and 40,000 pulses resulted in an ∼400-nm-thick film. The thin film sample was used for two reasons: (1) an edge-oriented substrate facilitates imaging along a low index zone axis perpendicular to the thinnest portion of the sample (this can be challenging for random powder specimens); and (2) by capping the thin film with a conductor, one can provide a conductive pathway to mitigate the sample charging that ultimately manifests in image drift. To do so, E1 films were coated with 50 nm of indium tin oxide (ITO) at room temperature using radio frequency-magnetron sputtering. Indium tin oxide is the preferred conductor as it is mechanically similar to a halide oxide and thus responds comparably to mechanical polishing.

Laser ablated samples were examined by four-circle diffraction to assess crystallinity and epitaxy. Supplementary Fig. 4 shows a theta–two theta and an omega scan for E1 prepared at 600 °C. The films are epitaxial to the MgO substrate (expected since the lattice mismatch is below 1%), and the mosaicity observed in the omega circle is consistent with that present in the MgO substrate. MgO substrates are known to have limited crystal quality (∼0.02° in omega) due to the flame-fusion technique used to grow them.

An Allied Multiprep polishing system is utilized to prepare a cross-sectional electron microscopy sample by wedge polishing technique^41^. To achieve electron transparency, the polished sample is ion milled with a Fischione Model 1050 Ion Mill while cooling with liquid nitrogen.

A JEOL 2000 S/TEM is used to collect selected area diffraction patterns from the E1 thin film. An aberration corrected FEI Titan G260–300 kV S/TEM equipped with an X-FEG source and an advanced Super-XTM EDS detector system is used to analyse the structure and chemistry of E1. The Titan is operated at 200 kV for HAADF STEM imaging and EDS mapping with the convergence semi-angle set to 15 mrad. The atomic-resolution EDS map indicating the position and the arrangement of the ions in the unit cell can be explained by corresponding HAADF–STEM images in which the atomic columns containing heavier elements are observed brighter.

We note that STEM analysis is also performed on cryogenically fractured E1 powder samples, and epitaxial thin films along [001] and [110] zone axes. In all cases STEM EDS analysis revealed no second phases and homogeneous and random elemental distributions within the E1 crystals. The STEM data featured in Fig. 5 of the main text was chosen since the thin film configuration coated with a capping layer of ITO mitigated charging most effectively and allowed access to near-atomic resolution with channelling conditions.

Supplementary Fig. 5 is a selected area diffraction pattern for E1 taken along <001>, the pattern contains no diffraction events that are attributable to second phases or to cation ordering. As such, we conclude single phase on the local scale. Supplementary Fig. 6 is a lower magnification STEM image showing a wider area view as compared with STEM EDS data in the main text. Two observations are of particular note: (1) the HAADF-STEM image on the left suggests high crystallinity; and (2) the STEM–EDS analysis shows no evidence for chemical segregation or phase separation over a lager range.

### Configurational entropy in the ideal model

The following derivation describes the method to determine the composition dependence of configurational entropy shown in Fig. 2b of the main text. An *N*-species system having composition {*x_i_*} has ideal entropy equal to:


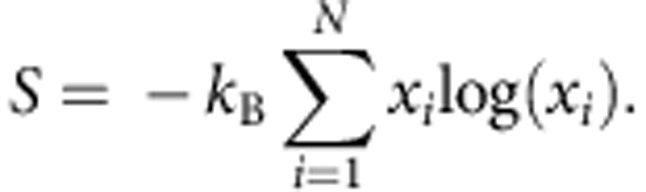


The maximum *S* is reached at equicomposition *x*_*i*_=1/*N* for each *i*, so:


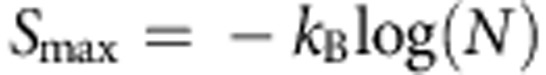


If only one species is varied, composition *x*_1_=*x* for instance, while leaving the other *N*−1 species at equicomposition:


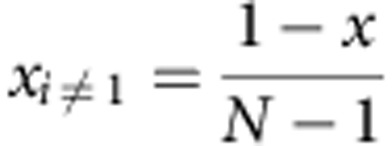


the ideal entropy becomes:


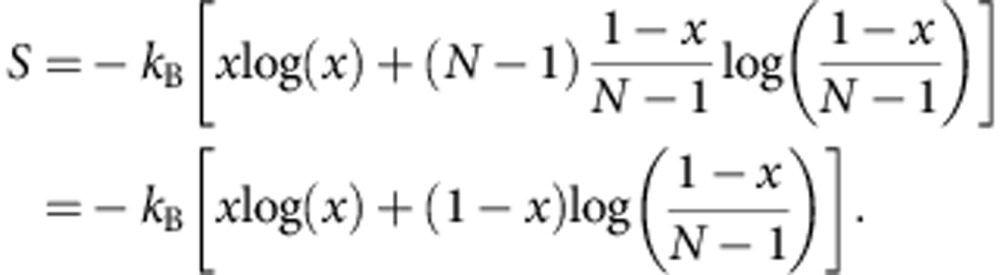


An expanded plot of entropy versus *N* for the entire series is shown in the Supplementary Fig. 7.

## Additional information

**How to cite this article:** Rost, C. M. *et al*. Entropy-stabilized oxides. *Nat. Commun.* 6:8485 doi: 10.1038/ncomms9485 (2015).

## Supplementary Material

Supplementary Information Supplementary Figures 1-7 and Supplementary Tables 1-4
