# Supplementary material for: Entropy-stabilized oxides
Source: Nat Commun. 2015 Sep 29;6:8485. doi: 10.1038/ncomms9485 (PMC4598836; doi:10.1038/ncomms9485)
Supplement: Supplementary Information — Supplementary Figures 1-7 and Supplementary Tables 1-4 [file ncomms9485-s1.pdf]

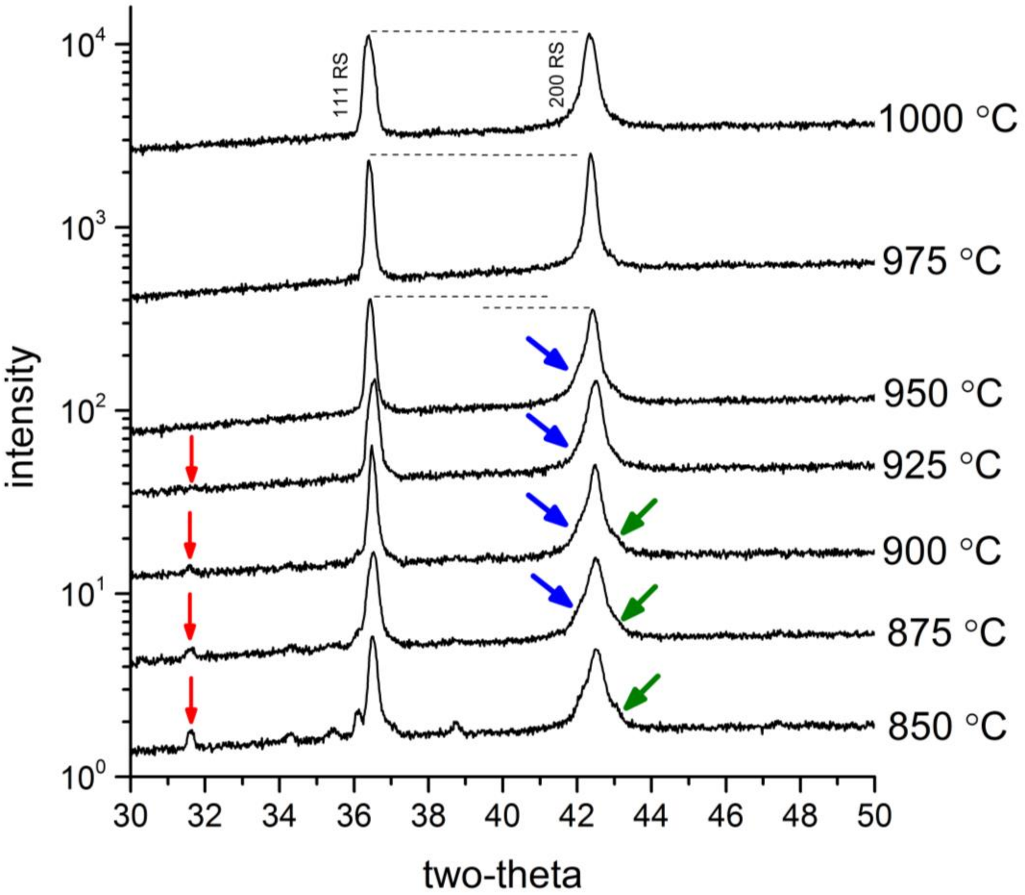

SUPPLEMENTARY Figure 1: Expanded view of one XRD temperature ladder showing the progression to single phase with 25 °C annealing steps. Red arrows indicate second phases, green and blue arrows indicate shoulders of second phases associated with wurtsitic and rocksalt structures respectively. Dashed lines highlight relative intensity values. Note that for rocksalt, I_{111} cannot be larger than I_{200}, thus we can use the relative intensity values as a co-indicator of the final transition to single phase.

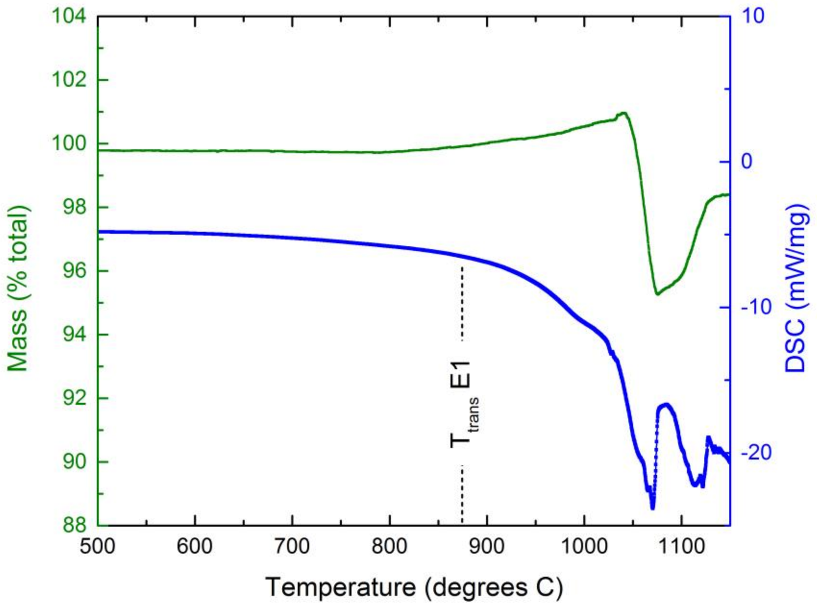

SUPPLEMENTARY Figure 2: DSC and TGA analysis for pure CuO powder collected under the identical conditions as sample E1, 25 °C/minute ramp in flowing air.

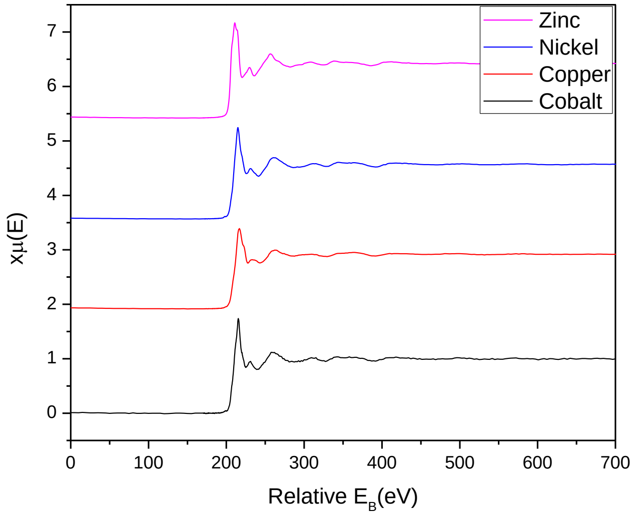

SUPPLEMENTARY Figure 3: Relative XAFS spectra as measured at APS beamlime 12-BM after energy normalization.

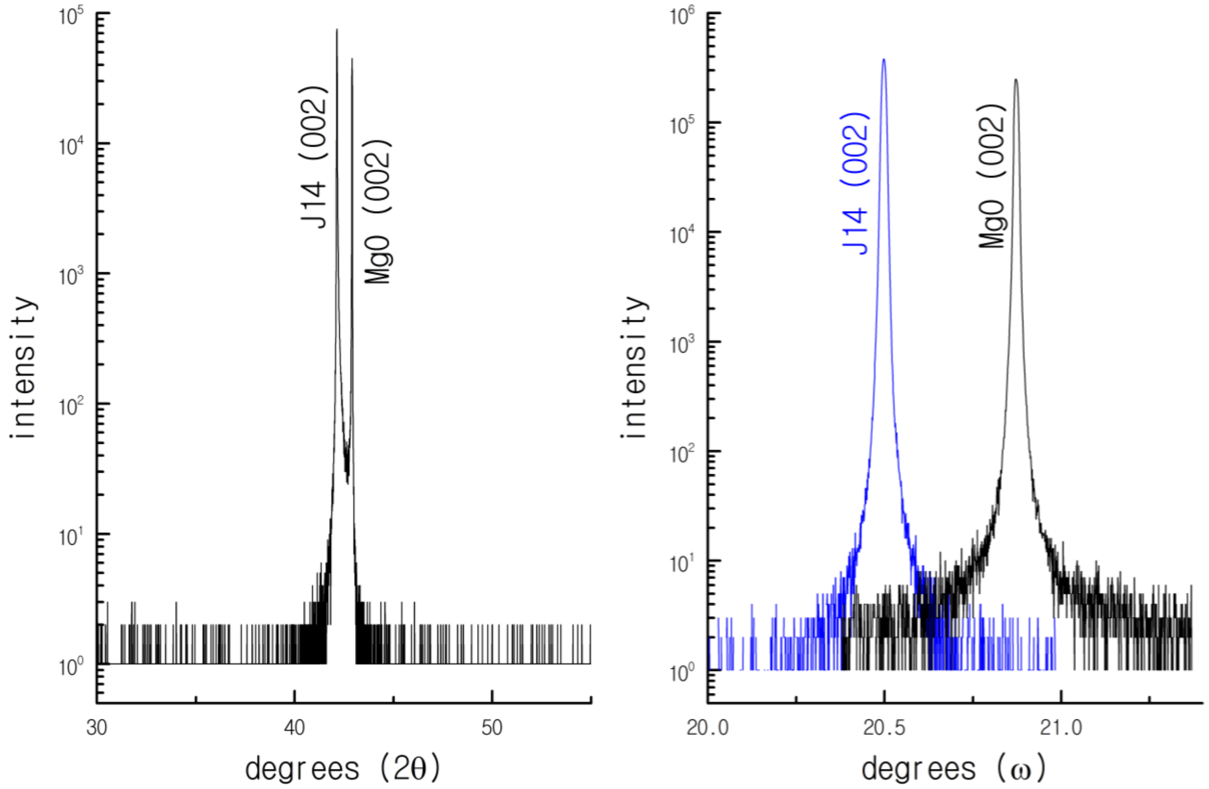

SUPPLEMENTARY Figure 4: X-ray analysis in the (left) two-theta and (right) omega circles for a {001} E1 film grown on an MgO single crystal substrate. The rocking curves were collected at the optimized 2θ positions for the film and substrate respectively. Both rocking curves have full-width-half-maximum values of 0.017°.

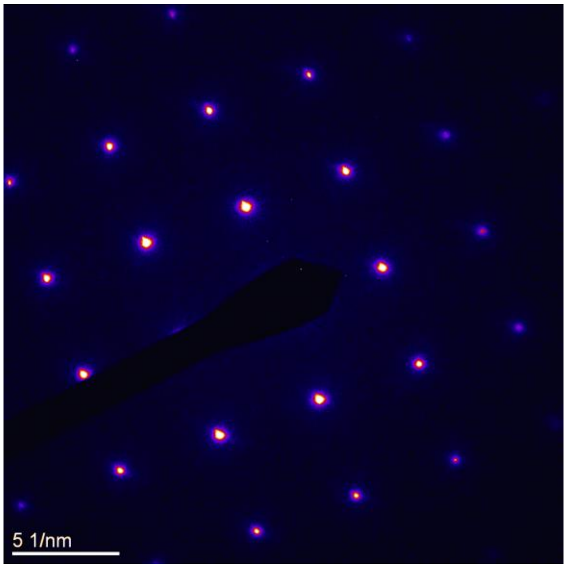

SUPPLEMENTARY Figure 5: False colored selected area electron diffraction pattern for formulation E1 taken along a <001> direction. There is no evidence for ordering or second phases..

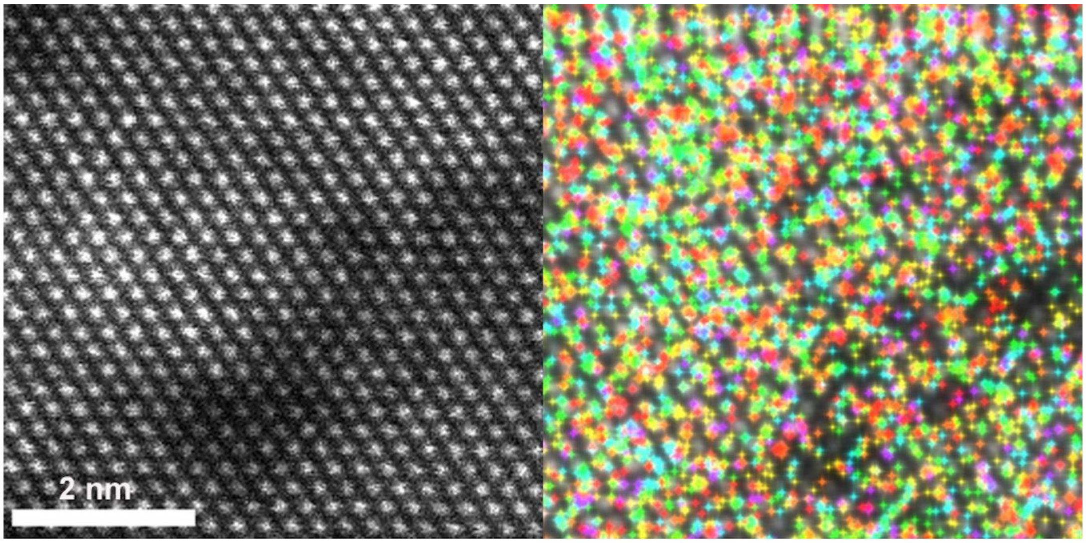

SUPPLEMENTARY Figure 6: A lower magnification HAADF STEM image of formulation E1 (left) and a composite STEM EDS map (right). In this map, the colors correspond to characteristic x-ray intensities where blue=Mg, purple=Co, green=Ni, orange=Cu, and red=Zn. Note that the areal distribution of colors is random and free from clustering.

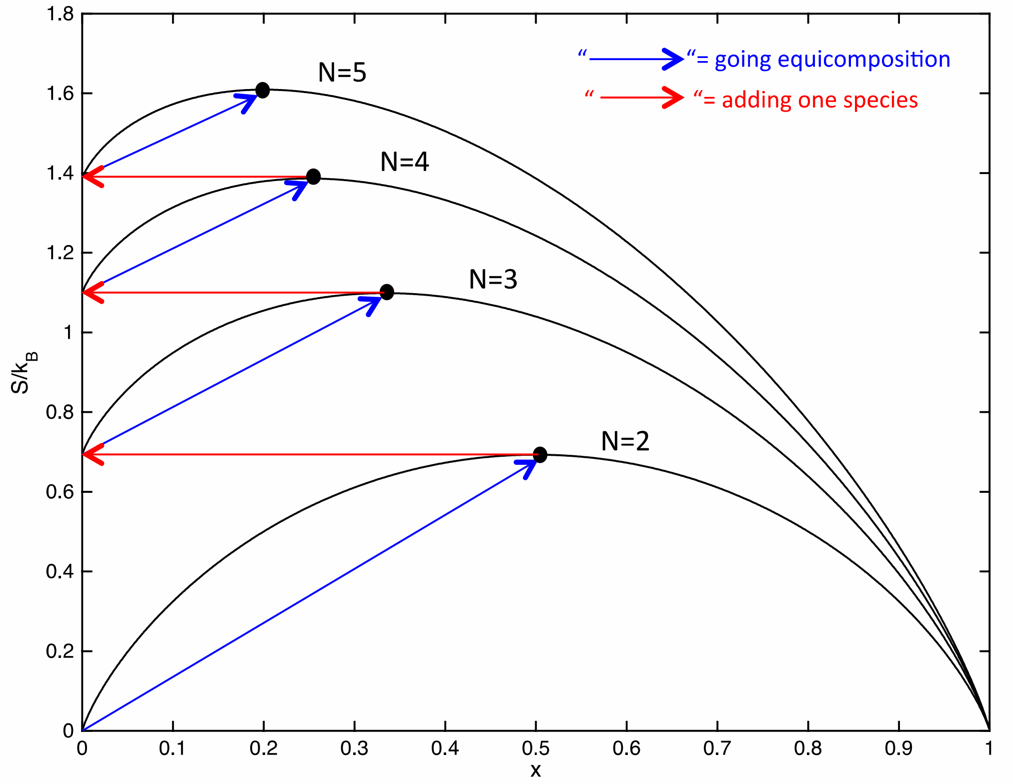

SUPPLEMENTARY Figure 7: Calculated configurational entropy in an *N*-component solid solutions as a function of Mol% of the Nth component. The black dots indicate the compositions with maximum configurations, and for each *N* value occur at the equimolar elemental ratios. The same calculation produced the data in Figure 2(b).

SUPPLEMENTARY TABLE 1: INITIAL OXIDE COMPONENTS IN ALLOY E1

**Binary Oxide**	**Structure (Space Group)**	**N**	**Cation Radius (nm)**
MgO	Rocksalt (Fm-3m)	6	0.072
NiO	Rocksalt (Fm-3m)	6	0.063
CoO	Rocksalt (Fm-3m)	6	L-0.065 H-0.074*
CuO	Tenorite (C 2/c)	4	0.073
ZnO	Wurtzite ($P6_3mc$)	4	0.074

*L denotes low spin, H denotes high spin

From: R.D. Shannon and C.T. Prewitt, "Effective ionic radii in oxides and fluorides," Acta. Cryst., v. B25, p925, p. 5, (1969).

SUPPLEMENTARY TABLE 2: LIST OF COMPOSITIONS USED TO PREPARE THE TRANSITION TEMPERATURE PHASE DIAGRAMS IN FIGURES 2(C-G) IN THE MAIN TEXT

Sample Name	Composition (x = 0.2)
J14	$Mg_xNi_xCo_xCu_xZn_xO$
J14K-10	$Mg_{(x+0.025)}Ni_{(x+0.025)}Co_{(x+0.025)}Cu_{(x+0.025)}Zn_{(x-0.1)}O$
J14K-6	$Mg_{(x+0.015)}Ni_{(x+0.015)}Co_{(x+0.015)}Cu_{(x+0.015)}Zn_{(x-0.06)}O$
J14K-2	$Mg_{(x+0.005)}Ni_{(x+0.005)}Co_{(x+0.005)}Cu_{(x+0.005)}Zn_{(x-0.02)}O$
J14K2	$Mg_{(x-0.005)}Ni_{(x-0.005)}Co_{(x-0.005)}Cu_{(x-0.005)}Zn_{(x+0.02)} O$
J14K6	$Mg_{(x-0.015)}Ni_{(x-0.015)}Co_{(x-0.015)}Cu_{(x-0.015)}Zn_{(x+0.06)}O$
J14K10	$Mg_{(x-0.025)}Ni_{(x-0.025)}Co_{(x-0.025)}Cu_{(x-0.025)}Zn_{(x+0.1)}O$
J14L-10	$Mg_{(x-0.1)} Ni_{(x+0.025)}Co_{(x+0.025)}Cu_{(x+0.025)}Zn_{(x+0.025)}O$
J14L-6	$Mg_{(x-0.06)} Ni_{(x+0.015)}Co_{(x+0.015)}Cu_{(x+0.015)}Zn_{(x+0.015)}O$
J14L-2	$Mg_{(x-0.02)} Ni_{(x+0.005)}Co_{(x+0.005)}Cu_{(x+0.005)}Zn_{(x+0.005)}O$
J14L2	$Mg_{(x+0.02)} Ni_{(x-0.005)}Co_{(x-0.005)}Cu_{(x-0.005)}Zn_{(x-0.005)}O$
J14L6	$Mg_{(x+0.06)} Ni_{(x-0.015)}Co_{(x-0.015)}Cu_{(x-0.015)}Zn_{(x-0.015)}O$
J14L10	$Mg_{(x+0.1)} Ni_{(x-0.025)}Co_{(x-0.025)}Cu_{(x-0.025)}Zn_{(x-0.025)}O$
J14M-10	$Mg_{(x+0.025)}Ni_{(x-0.1)}Co_{(x+0.025)}Cu_{(x+0.025)}Zn_{(x+0.025)}O$
J14M-6	$Mg_{(x+0.015)}Ni_{(x-0.06)}Co_{(x+0.015)}Cu_{(x+0.015)}Zn_{(x+0.015)}O$
J14M-2	$Mg_{(x+0.005)}Ni_{(x-0.02)}Co_{(x+0.005)}Cu_{(x+0.005)}Zn_{(x+0.005)}O$
J14M2	$Mg_{(x-0.005)}Ni_{(x+0.02)}Co_{(x-0.005)}Cu_{(x-0.005)}Zn_{(x-0.005)}O$
J14M6	$Mg_{(x-0.015)}Ni_{(x+0.06)}Co_{(x-0.015)}Cu_{(x-0.015)}Zn_{(x-0.015)}O$
J14M10	$Mg_{(x-0.025)}Ni_{(x+0.1)}Co_{(x-0.025)}Cu_{(x-0.025)}Zn_{(x-0.025)}O$
J14N-10	$Mg_{(x+0.025)}Ni_{(x+0.025)}Co_{(x-0.1)}Cu_{(x+0.025)}Zn_{(x+0.025)}O$
J14N-6	$Mg_{(x+0.015)}Ni_{(x+0.015)}Co_{(x-0.06)}Cu_{(x+0.015)}Zn_{(x+0.015)}O$
J14N-2	$Mg_{(x+0.005)}Ni_{(x+0.005)}Co_{(x-0.02)}Cu_{(x+0.005)}Zn_{(x+0.005)}O$
J14N2	$Mg_{(x-0.005)}Ni_{(x-0.005)}Co_{(x+0.02)}Cu_{(x-0.005)}Zn_{(x-0.005)}O$
J14N6	$Mg_{(x-0.015)}Ni_{(x-0.015)}Co_{(x+0.06)}Cu_{(x-0.015)}Zn_{(x-0.015)}O$
J14N10	$Mg_{(x-0.025)}Ni_{(x-0.025)}Co_{(x+0.1)}Cu_{(x-0.025)}Zn_{(x-0.025)}O$
J14O-10	$Mg_{(x+0.025)}Ni_{(x+0.025)}Co_{(x+0.025)}Cu_{(x-0.1)}Zn_{(x+0.025)}O$
J14O-6	$Mg_{(x+0.015)}Ni_{(x+0.015)}Co_{(x+0.015)}Cu_{(x-0.06)}Zn_{(x+0.015)}O$
J14O-2	$Mg_{(x+0.005)}Ni_{(x+0.005)}Co_{(x+0.005)}Cu_{(x-0.02)}Zn_{(x+0.005)}O$
J14O2	$Mg_{(x-0.005)}Ni_{(x-0.005)}Co_{(x-0.005)}Cu_{(x+0.02)}Zn_{(x-0.005)}O$
J14O6	$Mg_{(x-0.015)}Ni_{(x-0.015)}Co_{(x-0.015)}Cu_{(x+0.06)}Zn_{(x-0.015)}O$
J14O10	$Mg_{(x-0.025)}Ni_{(x-0.025)}Co_{(x-0.025)}Cu_{(x+0.1)}Zn_{(x-0.025)}O$

SUPPLEMENTARY TABLE 3: CALCULATED RELATIVE INTENSITY RATIOS FOR E1

Peak	hkl	d(Å)	2θ(°)	I/Imax
1	111	2.423	37.073	67.0%
2	200	2.100	43.073	100.0%
3	220	1.485	62.550	57.6%
4	311	1.266	74.998	26.4%

SUPPLEMENTARY TABLE 4: ELEMENTS MEASURED AT APS BEAMLINE 12-BM-B

Element of Interest	Reference Material	K-edge Energy (eV)
Cobalt	Co Metal Foil	7709
Nickel	Ni Metal Foil	8333
Copper	Cu Metal Foil	8979
Zinc	Zn Metal Foil	9659
